# Tunable Silver-Functionalized Porous Frameworks for Antibacterial Applications

**DOI:** 10.3390/antibiotics7030055

**Published:** 2018-07-03

**Authors:** Mark A. Isaacs, Brunella Barbero, Lee J. Durndell, Anthony C. Hilton, Luca Olivi, Christopher M. A. Parlett, Karen Wilson, Adam F. Lee

**Affiliations:** 1 Department of Chemistry, University College London, London WC1H 0AJ, UK; mark.isaacs@ucl.ac.uk; 2 European Bioenergy Research Institute, Aston University, Birmingham B4 7ET, UK; brubarbero@gmail.com (B.B.); chrisparlett81@gmail.com (C.M.A.P.); 3 Inorganic Chemistry and Catalysis, Debye Institute for Nanomaterials Science, Utrect University, Universiteitsweg 99, 3584 CG Utrecht, The Netherlands; l.j.durndell@uu.nl; 4 Life and Health Sciences, Aston University, Birmingham B4 7ET, UK; a.c.hilton@aston.ac.uk; 5 Sincrotrone Trieste, 34149 Basovizza, Italy; luca.olivi@elettra.eu; 6 School of Science, RMIT University, Melbourne, VIC 3001, Australia

**Keywords:** silver, antibacterial, titania, mesoporous, macroporous, surface functionalization

## Abstract

Healthcare-associated infections and the rise of drug-resistant bacteria pose significant challenges to existing antibiotic therapies. Silver nanocomposites are a promising solution to the current crisis, however their therapeutic application requires improved understanding of underpinning structure-function relationships. A family of chemically and structurally modified mesoporous SBA-15 silicas were synthesized as porous host matrices to tune the physicochemical properties of silver nanoparticles. Physicochemical characterization by transmission electron microscopy (TEM), X-ray diffraction (XRD), X-ray photoelectron spectroscopy (XPS), X-ray absorption near-edge spectroscopy (XANES) and porosimetry demonstrate that functionalization by a titania monolayer and the incorporation of macroporosity both increase silver nanoparticle dispersion throughout the silica matrix, thereby promoting Ag_2_CO_3_ formation and the release of ionic silver in simulated tissue fluid. The Ag_2_CO_3_ concentration within functionalized porous architectures is a strong predictor for antibacterial efficacy against a broad spectrum of pathogens, including *C. difficile* and methicillin-resistant Staphylococcus aureus (MRSA).

## 1. Introduction

Healthcare-associated infections (HCAIs) impact on millions of patients annually, and hundreds of thousands of deaths worldwide [1,2,3,4]. Current models predict that global mortality rates will approach 10 million annual deaths as a direct result of HCAIs by 2050 [5]. Approximately one-third of all such HCAIs are preventable [6], and while recent improvements in hygiene standards across the medical sector have for example significantly reduced the number of HCAIs per year in the UK, certain groups remain at very high risk of infection during hospital visits, and infection rates for some bacteria such as *Escherichia coli* have even increased [7,8,9].

The rise of drug-resistant bacteria which are disproportionately responsible for HCAIs, such as methicillin-resistant *Staphylococcus aureus* (MRSA) and extended-spectrum beta-lactamases (ESBL)-producing *Enterobacteriaceae* [2], has prompted a resurgence in the search for broad-spectrum antibiotics to tackle HCAIs. Silver is one such broad spectrum antibiotic, effective against Gram-positive (e.g., MRSA, *B. subtilis*) and Gram-negative bacteria (e.g., *E. coli*, *P. aeruginosa*) [10], and is consequently the subject of commercial [11] and academic interest for the treatment of existing HCAIs and drug-resistant organisms through its incorporation in surface coatings [12], nanotechnology [13,14,15,16], and pharmaceuticals [17,18,19].

A major advantage of nanoparticulate silver (and silver-based solid compounds) compared with salts or complexes is that oxidative dissolution is confined to exposed surfaces, offering a potential route to regulate the release of biologically active (commonly held to be Ag^+^) [20] species permitting the design of long lasting antimicrobial therapies [15,21,22,23]. Control over silver dissolution kinetics in Ag-nanoparticle-(NP) based systems has been investigated through tuning particle size [23,24,25,26], the use of inert coatings to retard release [26], or control of silver speciation in nanocomposite to accelerate dissolution and hence antibacterial activity [13,14]. Silver has also shown recent promise as a promoter of conventional antibiotic therapies [27].

Here we explore the use of high surface area and porous metal oxide frameworks to control the dispersion and subsequent dissolution of surface silver. The impact of framework porosity and oxide termination on silver speciation, dissolution, and antibacterical efficacy, was studied using ordered mesoporous and hierarchical macroporous-mesoporous SBA-15 silicas [28,29], and titania functionalized analogues. Conformal titania surface coatings, and hierarchically porous architectures promote silver NP dissolution and activity against Gram-positive (*Staphylococcus aureus*) and Gram-negative (*Pseudomonas aeruginosa*) bacteria.

## 2. Results and Discussion

### 2.1. Materials Synthesis and Characterisation

**Figure 1:**
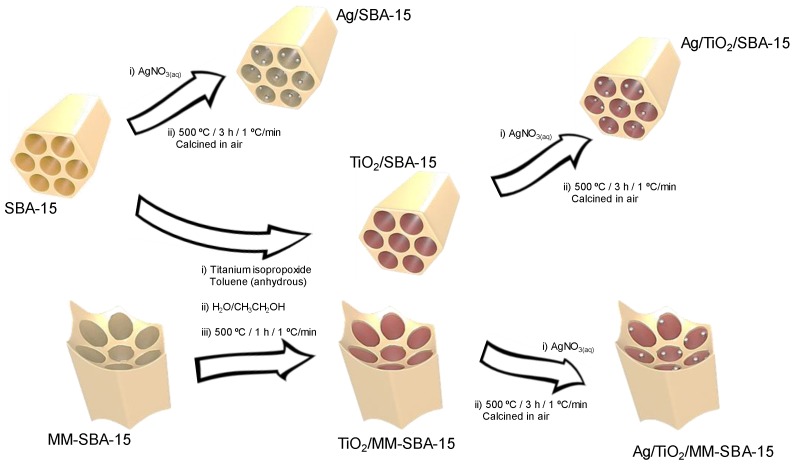
Synthesis of Ag-doped mesoporous silica and titania-functionalized mesoporous and mesoporous-macroporous silica materials.

Three families of ordered porous frameworks were prepared using soft or hard-soft dual templating approaches and subsequent titania functionalization (Figure 1). Mesoporous and macroporous-mesoporous (MM) SBA-15 silicas were prepared using a Pluronic 123 (P123) surfactant either alone or in conjunction with 400 nm colloidal polystyrene nanospheres respectively, according to literature methods. Titania modified variants were subsequently prepared adapting the procedure of Landau et al. [30] to preactivate surface silanols before the self-limited grafting of titanium isopropoxide under anhydrous conditions, and calcination. Repeated grafting cycles were used to create conformal TiO_2_ monolayers encapsulating the silica supports. Silver nanoparticles were introduced to either the parent SBA-15, or TiO_2_-grafted SBA-15 and TiO_2_-grafted MM-SBA-15, frameworks by wet impregnation with varying silver nitrate concentrations.

**Figure 2:**
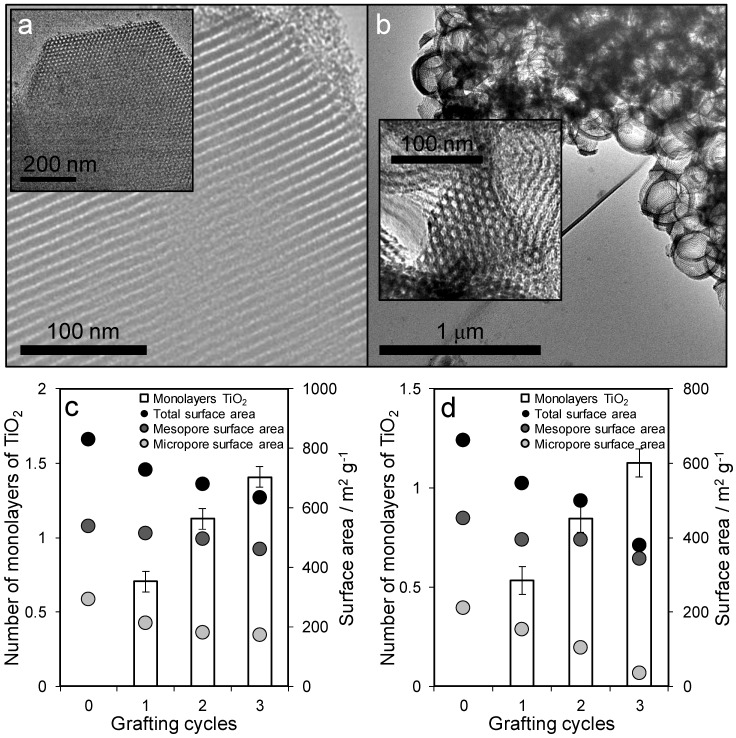
Bright-field transmission electron microscopy (TEM) images of (**a**) TiO_2_/SBA-15 (3^rd^ cycle grafting), and (**b**) TiO_2_/MM-SBA-15 (3^rd^ cycle grafting) highlighting ordered hexagonally close-packed mesopores and uniform macropores, and the evolution of textural properties and calculated TiO_2_ monolayer thickness as a function of grafting cycle for (**c**) TiO_2_/SBA-15 and (**d**) TiO_2_/MM-SBA-15.

Textural properties of the parent SBA-15 and MM-SBA-15 supports, before and after titania functionalization, were first characterized by transmission electronic microscopy (TEM), N_2_ porosimetry, X-ray diffraction (XRD), and X-ray photoelectron spectroscopy (XPS). The expected hexagonally close-packed channels of the SBA-15 mesopore network were observed in all cases, with surface areas and mesopore diameters decreasing with successive TiO_2_ grafting cycle (Figure 2 and Figure S1), reflecting pore narrowing and partial blockage of micropores in the silica framework [31]. Uniform mesopores of 6.3 nm diameter were in accordance with previous reports [29]. No titania crystallites were visible by TEM following grafting, indicating the formation of highly dispersed phase. Assuming a TiO_2_ monolayer thickness of 0.355 nm [32], mesopore shrinkage across three grafting cycles from 6.3 to 5.3 nm for SBA-15 was consistent with deposition of a 1.4 monolayer (ML) titania coating, and from 6 to 5.2 nm for MM-SBA-15 consistent with 1.1 ML titania. 

**Figure 3:**
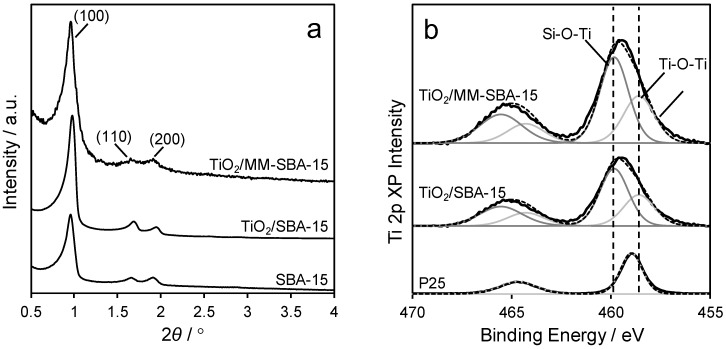
(**a**) Low angle X-ray diffraction (XRD), and (**b**) fitted Ti 2p XP spectra of TiO_2_/SBA-15 and TiO_2_/MM-SBA-15, alongside pure silica (SBA-15) and titania (P25) references.

Low-angle X-ray diffraction (XRD) (Figure 3) confirmed that titania-functionalized frameworks retained the *P6mm* symmetry of the parent SBA-15 mesopore network. Wide angle XRD (Figure S2) showed no evidence for crystalline titania phases, consistent with TEM and the formation of conformal monolayers over the SBA-15 template. The surface chemical environment of Ti atoms within the monolayer coatings was examined by XPS, which reveals the presence of Ti-O-Si (associated with the silica-titania interface) and Ti-O-Ti species over both SBA-15 and MM-SBA-15 with Ti 2p_5/2_ binding energies of 459.9 eV and 458.5 eV respectively. The shift to higher binding energy for Ti coordinated to Si (through a bridging oxygen) reflects the higher electronegativity of the latter, and hence higher induced initial charge on the former. We attribute the small shift between the Ti-O-Ti environments in P25 and the titania monolayers to quantum size (initial and/or final state) effects. 

Silver was subsequently deposited over the unfunctionalized SBA-15, and TiO_2_/SBA-15 and TiO_2_/MM-SBA-15 at three different nominal loadings spanning 0.3–3 wt% (see the Supplementary Materials (Table S1); elemental analysis revealed almost identical bulk loadings for the mesoporous materials, but systematically lower loadings for the hierarchical material. Surface and bulk silver loadings were generally in good agreement for all materials (see the Supplementary Materials Table S1), indicating a homogenous distribution throughout the porous frameworks. Physicochemical properties of the resulting silver species were determined by TEM, XPS, XRD, and X-ray absorption near edge spectroscopy (XANES). Only fcc metallic silver (JCPDS no. 04-0783) reflections were observed by XRD, which sharpened with increasing loading (see the Supplementary Materials Figure S3), indicative of crystallite growth. Volume averaged silver NP sizes were smaller for MM-SBA-15 (3.5–6.5 nm) than SBA-15 (4.5–8.5 nm) at comparable loadings (~0.3 wt%), evidencing higher metal dispersion over the hierarchically porous framework, and indeed fell below the threshold of detection for the lowest 0.25 wt% Ag/TiO_2_/MM-SBA-15. Ag NPs were directly visualized by TEM (see the Supplementary Materials Figures S4–S6), which revealed a relatively broad size distribution spanning 1–20 nm over all three supports. The mean particle size increased (and size distribution narrowed) with Ag loading (see the Supplementary Materials Figure S7), with the smallest NPs observed over the hierarchical support in all cases, in accordance with XRD. Ag 3d XP spectra showed the existence of two distinct chemical environments with 3d_5/2_ spin-orbit split component binding energies of 368.0 eV and 370.5 eV consistent with Ag^0^ and possibly a surface Ag_2_CO_3_ respectively (see the Supplementary Materials Figure S8) [33].

**Figure 4:**
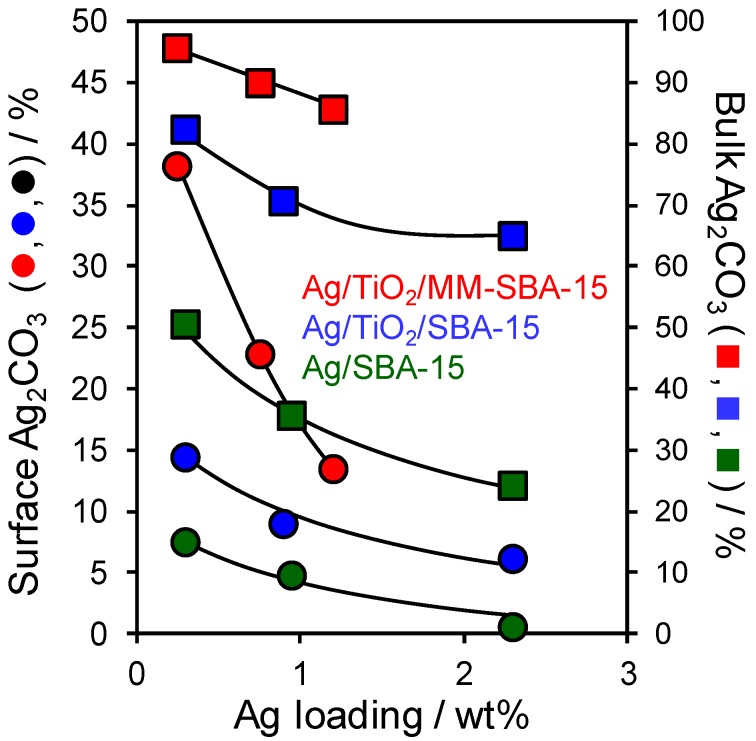
Surface (XPS) and bulk (XANES) silver speciation as Ag_2_CO_3_ of Ag/SBA-15, Ag/TiO_2_/SBA-15, and Ag/TiO_2_/MM-SBA-15 as a function of Ag loading.

Note that discrimination of silver oxides and carbonate by XPS alone is complicated due to their similar core-level binding energies, and anomalous negative binding energy shift of the oxides relative to the metal [14,34,35] and therefore requires careful fitting to well-defined standards (difficult to guarantee due to the tendency of Ag_2_O to oxidise, and of both oxides to adsorb atmospheric CO_2_ and form surface carbonates [36]), or Auger parameter analysis employing the Ag M_4,5_NN Auger transition in conjunction with 3d_5/2_ core-level spectra [13]. Silver speciation as metal, oxide, or carbonate was therefore examined by linear combination fitting of Ag K-edge X-ray absorption near-edge spectroscopy (XANES, see the Supplementary Materials Figures S9–S11). In all cases, good spectral fits could only be obtained using Ag and Ag_2_CO_3_ components. Nanoparticle growth is accompanied by a continuous decrease in the proportion of surface and bulk silver carbonate for all frameworks (Figure 4). This switchover from electron deficient to metallic silver with particle size likely reflects the higher surface energy of the latter [37], which is thus favoured for larger particles. The bulk Ag_2_CO_3_ content determined by XANES was systematically higher than that of the surface determined by XPS, likely reflecting the distribution of particle sizes present in all materials; XPS is expected to more sensitive to larger (more metallic) particles preferentially located on the external surface. AgO and Ag_2_O could not be detected by linear combination fitting of the XANES data. 

### 2.2. Materials Performance Assaying

**Figure 5:**
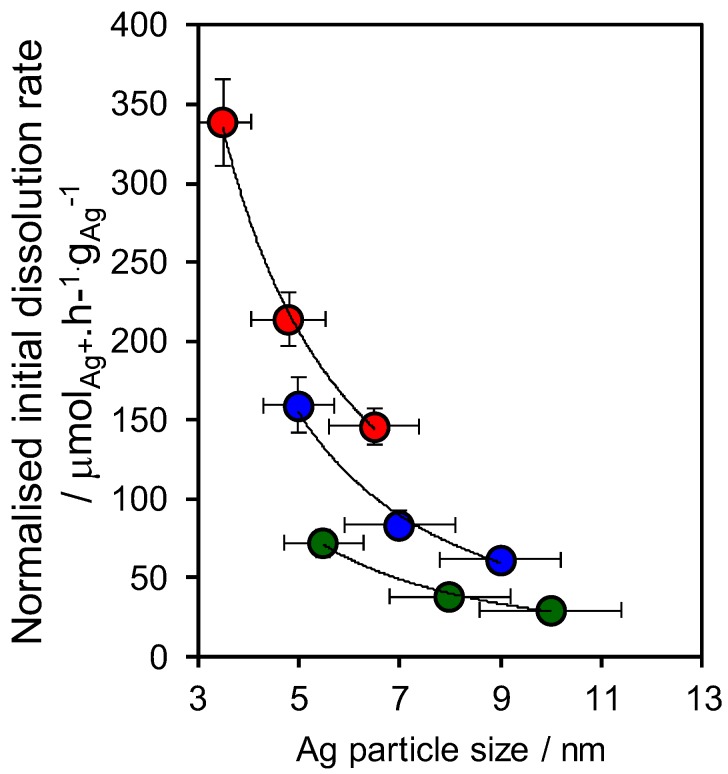
Ag^+^ dissolution rates normalized to mass of Ag determined by XANES for Ag/SBA-15, Ag/TiO_2_/SBA-15 and Ag/TiO_2_/MM-SBA-15.

The release rate of ionic silver (Ag^+^) from the preceding SBA-15 and MM-SBA-15 frameworks in a 0.5 M NaNO_3_ solution at 37 °C was subsequently determined by ICP-MS analysis (Figure 5). Dissolution rates normalised to the mass of silver were inversely proportional to particle size (loading) for all frameworks, as reported for citrate stabilized silver metal nanoparticles [23] and core-shell silver-silica nanoparticles [26] indicating that the release of ionic silver occurred by a common mechanism, being solely dependent on the geometric surface area of the silver nanoparticles. Coating of the mesoporous SBA-15 by a conformal titania monolayer slightly decreased the average particle size over the bare silica framework, and hence increased the rate of silver dissolution, a phenomenon further enhanced by the introduction of macropores. The rate constant for Ag^+^ dissolution was determined as 5244 μmol·h^−1^·nm^−1^·g^1^_Ag_ by fitting the data (see the Supplementary Materials Figure S12) in accordance with the method of Zhang et al. [23] This is significantly higher than that found for Ag@SiO_2_ core-shell nanoparticles (14.7 μmol·h^−1^·nm^−1^·g^−1^_Ag_) [26], while the absolute release rate of the 0.3 wt% Ag/TiO_2_/MM-SBA-15 is consistent with that previously observed for 0.05 wt% high surface area Ag-hydroxyapatite nanoparticles [14].

**Figure 6:**
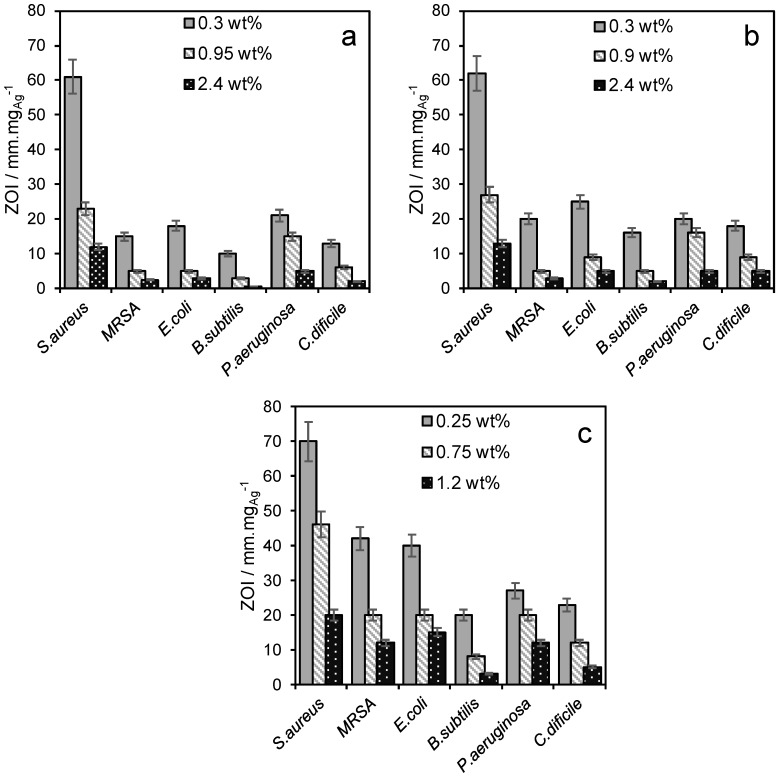
Zone of inhibition plots, normalized to mass of Ag, against a range of Gram-positive and Gram-negative bacteria for (**a**) Ag/SBA-15, (**b**) Ag/TiO_2_/SBA-15 and (**c**) Ag/TiO_2_/MM-SBA-15.

Antibacterial activity was subsequently assessed against a range of bacteria (*Pseudomonas aeruginosa*, *Staphylococcus aureus*, *Clostridium difficile*, *Escherichia coli*, *Bacillus subtilis* and MRSA) by zone plate inhibition (ZoI) (Figure 6) using simulated body fluid (SBF), in which the antimicrobial efficacy is proportional to the size of the colony-free zone surrounding the silver functionalized porous frameworks. None of the parent (silver-free) materials exhibited bactericidal properties. In all cases the ZoI values normalized to the mass of silver were inversely proportional to silver loading, mirroring the ionic silver release rates, and superior to those reported for hydroxyapatite (HA) supported Ag_3_PO_4_ composites or Ag@SiO_2_ core-shell nanocomposites. For example, for *S. aureus* Ag/TiO_2_/MM-SBA-15 achieved 70 mm·g_Ag_^−1^ versus 40–60 mm·g_Ag_^−1^ for a high area Ag-HA [14] or 23 mm·g_Ag_^−1^ for 4.5 nm Ag nanoparticles encapsulated by a 10.5 nm mesoporous silica shell) [26]. The zone size (antibacterial performance) decreased in the sequence Ag/TiO_2_/MM-SBA-15 > Ag/TiO_2_/SBA-15 > Ag/SBA-15. 

**Figure 7:**
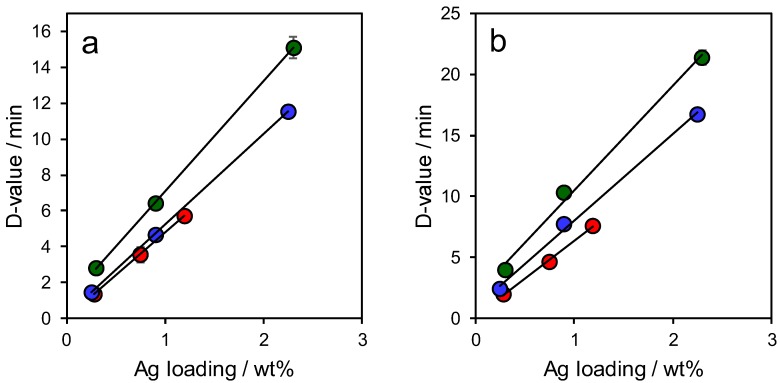
Decimal reduction times for Ag/TiO_2_/MM-SBA-15, Ag/TiO_2_/SBA-15 and Ag/SBA-15 against (**a**) *Staphylococcus aureus*, and (**b**) *Pseudomonas aeruginosa.*

The logarithmic reduction method was also employed to obtain a more quantitative evaluation of antibacterial activity for representative Gram-positive and Gram-negative bacteria (*Staphylococcus aureus* and *Pseudomonas aeruginosa* respectively). Experiments were performed in the absence of light to eliminate any possible influence from photogenerated reactive oxygen species by the semiconducting titania coating. All three frameworks were inactive in the absence of silver (see the Supplementary Materials Figure S13). Corresponding decimal reduction times (D-values), i.e., the time to kill 90% of the bacteria, normalised to the mass of silver, also evidenced an inverse relationship with silver loading for all frameworks (Figure 7) consistent with their Ag^+^ release rates. (Figures S14–S15). As for the ZoI assays, antibacterial activity followed the order Ag/TiO_2_/MM-SBA-15 > Ag/TiO_2_/SBA-15 > Ag/SBA-15, i.e., titania functionalization, and the introduction of macroporosity, both enhanced the potency of silver nanoparticles dispersed throughout an SBA-15 framework. Ag/MM-TiO_2_/SBA-15 outperformed Ag/SBA-15 by ~105% against *Staphylococcus aureus* and ~103% against *Pseudomonas aeruginosa*. The present observations are consistent with previous reports of augmented antimicrobial efficacy arising from the preferential formation of highly soluble silver carbonate versus silver metal over γ-alumina supports [13].

## 3. Conclusions

Families of silver functionalized porous frameworks based around surfactant templated SBA-15 mesoporous silica were synthesized to investigate the impact of surface chemistry and pore architecture on antibacterial performance. Silver nanoparticles introduced by wet impregnation of AgNO_3_ and subsequent thermal processing comprised mixed metal and carbonate phases, with the concentration of silver carbonate (and dissolution rate for ionic Ag^+^) inversely proportional to particle size independent of the framework. The introduction of a conformal titania monolayer coating of SBA-15 improved the dispersion of silver nanoparticles, corresponding bulk and surface carbonate content, and hence ionic silver release kinetics. This observation likely reflects the ability of defective reducible metal oxides to act as nucleation centers for metals [38,39,40], thereby increasing the density of small nanoparticles, and amphoteric nature of titania and ability to capture atmospheric CO_2_ [41] which can subsequently react with (weakly basic) silver oxides [42,43] at the nanoparticle interface. Additional rate enhancements for ionic silver were observed for hierarchical microporous-mesoporous SBA-15 frameworks, presumably due to their stabilization of even smaller silver nanoparticles and hence higher carbonate concentrations. Silver release rates from the porous frameworks directly correlated with broad spectrum antibacterial activity, with a 16–38% decrease in decimal reduction times for *Staphylococcus aureus* and *Pseudomonas aeruginosa* observed following titania functionalization, and a further 65–89% reduction following the introduction of macroporosity. The quantitative structure-function relationship identified between the concentration of Ag_2_CO_3_ and antibacterial efficacy will guide the development of future nanocomposite architectures, notably optimization of the reducible metal oxide coatings and pore networks to further promote ionic silver release.

## 4. Materials and Methods

Polystyrene bead templates were synthesized using a method developed by Vaudreuil et al. [44] in which styrene, divinyl benzene (comonomer, Sigma Aldrich, 80%) and potassium persulphate (initiator, Sigma Aldrich, >99%) were the reagents. The reaction was performed on a large scale in a 2-liter jacketed Radleys Reactor-Ready system at 90 °C. Deionized water (1.5 L) was introduced to the reactor, along with a Leibig condenser, thermocouple, and a nitrogen line at 1.5 bar pressure. The reactor was stirred at 300 rpm overnight to outgas the solution. Styrene (140 mL, Sigma Aldrich, >99%) and divinylbenzene (27 mL) were washed with NaOH (0.1 M) three times in separate separating funnels and added to the reaction vessel. Potassium persulfate (Sigma Aldrich, 0.35 g) was dissolved in deionized water (20 mL) at 80 °C. After 30 min of stirring (300 rpm) in the reactor at 90 °C, the potassium persulfate solution was added. After stirring for 3 h, the solid particles were recovered as a concentrated solution and stored in a freezer overnight, then the product was allowed to warm before being filtered, washed with ethanol and the beads dried at 80 °C overnight.

SBA-15 was prepared using a cooperative self-assembly method [29], in which a 2.6 wt% solution of Pluronic P123 triblock copolymer (poly(ethylene glycol)-poly(propylene glycol)-poly(ethylene glycol) (Sigma Aldrich), in 1.6-M HCl solution was stirred (500 rpm) at 35 °C. Tetraethyl orthoxysilicate (Sigma Aldrich, 98%) was then added to the mixture, at a molar ratio of 60:1 [TEOS]:[P-123]. The mixture was aged at 80 °C for 24 h without stirring in a sealed container in an oven. The resultant solid material was filtered, then washed with ethanol before drying in air at 100 °C overnight. Removal of the P123 framework was performed by calcination at 500 °C in a muffle furnace for 6 h with a ramp rate of 1 °C/min. Macropores were introduced by the addition of polystyrene beads to the SBA synthesis (2.3.1) at a weight ratio of 5.3:1 [PS beads]:[TEOS] in the initial mixture.

The grafting of titania onto the surface of the prepared silica materials was done using a modified procedure by Landau et al. [30] in which triethylamine is used to activate the surface silanols on the silica and allow the reaction to proceed at lower temperatures. To ensure a uniform coating of TiO_2_, the reaction must be performed under completely dry conditions, due to the facile hydrolysis of the titania precursor, which will readily form large titania particles in the presence of water. The synthetic procedure involves mixing titanium isopropoxide (Sigma Aldrich,) in anhydrous toluene (Aldrich, water content <0.002%), adding triethylamine (Sigma Aldrich, >99%) and MM-SBA-15 or SBA-15 material whilst stirring at 85 °C for 6 h under nitrogen flow. The concentration of titanium isopropoxide was 145 g/L, the molar ratio between titanium isopropoxide and SBA-15 was fixed at 3.5 and the triethylamine: SBA-15 weight ratio at 1.5 on a scale of 5 g of SBA-15/MM-SBA-15. After the reaction, the solid was separated by filtration, washed with toluene (300 mL) and inserted in a 0.5 wt% water-ethanol solution (500 mL) under stirring for 24 h. The resultant solid was washed with ethanol, dried in air in an oven at 90 °C for 24 h, then calcined for 1 h at 250 °C, 1 h 400 °C and finally for 4 h at 500 °C all at 1 °C/min.

Ag NPs were deposited via wet impregnation using a solution of aqueous silver nitrate (99.9%, Sigma-Aldrich). A slurry of the silver precursor and support (10 mL of 5–25 μM + 1 g support) was stirred for 18 h at room temperature before heating to 50 °C. After 5 h, agitation was ceased and the solid aged at 50 °C for a further 24 h to yield a dry powder. Dried samples were calcined at 500 °C (ramp rate 1 °C/min) in static air for 3 h.

XRD patterns were recorded on either a PANalytical X’pertPro diffractometer fitted with an X’celerator detector and Cu Kα (1.54 Ǻ) source or a Bruker D8 Advance diffractometer fitted with a LynxEye high-speed strip detector and Cu Kα (1.54 Ǻ) source. Both instruments were calibrated against either Si (PANalytical, Malvern, UK) or SiO_2_ (Bruker, Billerica, MA, USA) standards. Low angle patterns were recorded for 2*θ* = 0.3–8° with a step size of 0.01°. Wide angle patterns were recorded for 2*θ* = 10–80° with a step size of 0.02°.

N_2_ adsorption isotherms were recorded using a Nova 4000 porosimeter (Quantachrome, Boynton Beach, FL, USA), before which the samples were thoroughly degassed under vacuum at 120 °C for 2 h. T-plot analysis was used to calculate microporosity. Data was analyzed using NOVAWin version 11 (Quantachrome, Boynton Beach, FL, USA).

XPS analysis was recorded using a Kratos Axis Hsi X-ray photoelectron spectrometer (Kratos Analytical Ltd., Manchester, UK) using a monochromated Al K_α_ (1486.6 eV) anode. Data was charge corrected to adventitious carbon at 284.6 eV and analyzed using CASAXPS version 2.3.15. Ag 3D peaks were fitted using a Doniach-Sunjic modified Gaussian-Lorentz peak shape and a doublet separation of 6 eV.

TEM analysis was performed using a JEOL 2100 microscope (JEOL Ltd., Tokyo, Japan) with a LaB_6_ source and HT of 180 kV. Particle sizing was performed using ImageJ 1.46r software (open source). Samples were prepared using a drop casting method in ethanol onto continuous carbon grids.

Dissolution rates were determined by stirring 10 mg of composite in 25 mL of 0.5 M NaNO_3_ at 37 °C with periodic aliquots of the analyte solution measured for silver content using an Agilent 6130B single Quad (ESI) ICP-MS (Agilent Technologies, Santa Clara, CA, USA) calibrated against a range of silver concentrations made by serial dilution of a 1000 ppm Ag in 1% HNO3 standard from Sigma-Aldrich. 

The antibacterial performance of all materials was evaluated against *Staphylococcus aureus* ATCC 6538, MRSA ATCC 33591, *Escherichia coli* NCTC 10418, *Bacillus subtilis* NCTC 8236, *Pseudomonas aeruginosa* ATCC 15442 and *Clostridium difficile* ATCC 9689, which are representative Gram-positive and Gram-negative problematic organisms found in hospital environments. Zone of inhibition (ZoI) tests were performed by inoculating the surface of a nutrient agar plate (Oxoid, Basingstoke, UK) with an excess volume (3 mL) of nutrient broth which had previously been inoculated and incubated to a cell density of ~108 colony-forming units (cfu)/mL as determined spectrophotometrically using a Perkin-Elmer Lambda 10 UV-Vis spectrophotometer. The liquid was manipulated by agitation to provide a confluent inoculum and the excess fluid removed to waste using a sterile pipette. Using a sterilized boring tool, 5 mm holes were then bored into the agar, and 100 μL of a solution of 10 mg of Ag nanocomposite in 5 mL of simulated body fluid (SBF, see the Supplementary Materials Table S2) dispensed into the borehole using a calibrated micropipette. SBF was prepared according to a method from Kokubo et al. [45] 750 mL of deionized water was stabilized at 37 °C with stirring, to this the following ions were added: NaCl (7.996 g, Sigma Aldrich >99%), NaHCO_3_ (0.35 g, Sigma Aldrich >99%), KCl (0.224 g, Sigma Aldrich >99%), K_2_HPO_4_·3H_2_O (0.228 g, Sigma Aldrich >99%), MgCl_2_ (0.305 g, Sigma Aldrich >99%), HCl (40 mL, 1 kmol/L, Fisher scientific 37%), CaCl_2_ (0.278 g, Sigma Aldrich >99%), Na_2_SO_4_ (0.071 g, Sigma Aldrich >99%) and (CH_2_OH)3CNH_2_ (6.057 g, Sigma Aldrich 99%). Finally, the pH was adjusted to 7.35 using HCl solution (1 kmol/L, Fisher scientific 37%). Plates were then incubated at 37 °C overnight, photographed, and calibrated zone areas determined using ImageJ software (open source).

Quantitative antimicrobial activity was determined by logarithmic reduction [13,14]. Here, 5 mg of Ag nanocomposite material was added to an Eppendorf tube kept in dark conditions containing 1 mL of either *S. aureus* or *P. aeruginosa* in a nutrient broth at concentrations of 107 cfu·mL^−1^. 100 μL aliquots of the resulting suspensions were subsequently removed at 0, 60, 240 min and 24 h, and added to a 1 mL solution of Tween 20 (Fisher, 1%), sodium dodecyl sulphate (Sigma-Aldrich, 0.4%) and sodium chloride (Sigma-Aldrich, 0.85%) in deionized water to neutralise any soluble silver species [13,14].

Each of the resulting neutralized solutions was serially diluted with phosphate buffered saline (PBS) prior to plating onto agar and incubation at 37 °C for 24 h. The experiments were all run with positive and negative controls of silver nitrate and without any nanocomposite respectively. After incubation, the number of colonies present on the agar was counted by visual inspection, and normalized relative to the initial colony count in the negative control at time t = 0 min to determine the logarithmic reduction of bacteria. All experiments were performed in triplicate, with mean values and standard deviations reported.

## Supplementary Materials

The following are available online at http://www.mdpi.com/2079-6382/7/3/55/s1, Figure S1: (left) N_2_ adsorption-desorption isotherms, and BJH pore size distributions for parent SBA-15, TiO_2_/SBA-15 and TiO_2_/MM-SBA-15. Type-IV isotherms with H1 hysteresis is characteristic of mesoporous SBA-15, Table S1: Elemental analysis of Ag/SBA-15, Ag/TiO_2_/SBA-15 and Ag/TiO_2_/MM-SBA-15, Figure S2: XRD patterns for the three prepared support materials, SBA-15, TiO_2_/SBA-15 and TiO_2_/MM-SBA-15, Figure S3: XRD patterns for (a) Ag/SBA-15; (b) Ag/TiO_2_/SBA-15; and (c) Ag/TiO_2_/MM-SBA-15 as a function of silver loading. All reflections associated with fcc silver metal., Figure S4: Particle size distributions from TEM for (a) 0.3 wt%; (b) 0.95 wt% and (c) 2.4 wt% Ag nanoparticles deposited on SBA-15, Figure S5: Particle size distributions from TEM (a) 0.3 wt%; (b) 0.9 wt% and (c) 2.4 wt% Ag nanoparticles deposited on TiO_2_/SBA-15, Figure S6: Particle size distributions from TEM (a) 0.25 wt%; (b) 0.75 wt% and (c) 1.2 wt% Ag nanoparticles deposited on TiO_2_/MM-SBA-15, Figure S6: Particle size distributions from TEM (a) 0.25 wt%, (b) 0.75 wt% and (c) 1.2 wt% Ag nanoparticles deposited on TiO2/MM-SBA-15, Figure S7: Mean particle size and standard deviation for Ag/SBA-15, Ag/TiO2/SBA-15, and Ag/TiO2/MM-SBA-15 as a function of bulk silver loading, Figure S8: Fitted Ag 3d XP spectra of (a) Ag/SBA-15; (b) Ag/TiO_2_/SBA-15; and (c) Ag/TiO_2_/MM-SBA-15 as a function of Ag loading. All spectra fitted to carbonate and metal spin-orbit doublets possessing common lineshapes and fixed binding energies, Figure S9: XANES profiles for (a) 0.3 wt%; (b) 0.95 wt% and (c) 2.4 wt% Ag/SBA-15, Figure S10: XANES profiles for (a) 0.3 wt%; (b) 0.9 wt% and (c) 2.4 wt% Ag/TiO_2_/SBA-15, Figure S11: XANES profiles for (a) 0.25 wt%; (b) 0.75 wt% and (c) 1.2 wt% Ag/TiO_2_/MM-SBA-15, Figure S12: Fitted dissolution kinetics for Ag/SBA-15, Ag/TiO_2_/SBA-15, and Ag/TiO_2_/MM-SBA-15 as a function of particle size, Figure S13: Zone of Inhibition assays for *Staphylococcus aureus*, MRSA, *Escherichia coli*, *Bacillus subtilis*, *Pseudomonas aeruginosa* and *Clostridium difficile* normalized to the mass of silver for (a) Ag/SBA-15; (b) Ag/TiO_2_/SBA-15; and (c) Ag/TiO_2_/MM-SBA-15, Figure S14: Logarithmic reductions for SBA-15, Ag/TiO_2_/SBA-15, and Ag/TiO_2_/MM-SBA-15 against (a) *Staphylococcus aureus* and (b) *Pseudomonas aeruginosa* after 24 h incubation, Figure S15: Logarithmic reductions for (a) Ag/SBA-15; (b) Ag/TiO_2_/SBA-15; and (c) Ag/TiO_2_/MM-SBA-15 against *Staphylococcus aureus* and *Pseudomonas aeruginosa*, Figure S16: Logarithmic colony forming units of (a) *S. aureus*; and (b) *P. aeruginosa* as a function of time for SBA-15, TiO_2_/SBA-15, and TiO_2_/MM-SBA-15, Table S1: Elemental analysis of Ag/SBA-15, Ag/TiO2/SBA-15 and Ag/TiO2/MM-SBA-15, Table S2: Ion concentrations in SBF solution.
