# Supplementary material for: Tunable Silver-Functionalized Porous Frameworks for Antibacterial Applications
Source: Antibiotics (Basel). 2018 Jul 3;7(3):55. doi: 10.3390/antibiotics7030055 (PMC6165165; doi:10.3390/antibiotics7030055)
Supplement: Supplementary file 1 [file antibiotics-07-00055-s001.docx]

Electronic supporting information

Tunable Ag functionalised porous frameworks for antibacterial applications

Mark A. Isaacs ^1^, Brunella Barbero^2^, Lee J. Durndell ^3^, Anthony C. Hilton ^4^, Luca Olivi ^5^, Christopher M. A. Parlett ^2^, Karen Wilson^6^ and Adam F. Lee ^6^.*

^1^ Department of Chemistry, University College London; mark.isaacs@ucl.ac.uk

^2^ European Bioenergy Research Institute, Aston University, Birmingham, B4 7ET, UK.

^3^ Inorganic Chemistry and Catalysis, Debye Institute for Nanomaterials Science, Utrect University. Universiteitsweg 99, 3584 CG Utrect, The Netherlands.

^4^ Life and Health Sciences, Aston University, Birmingham, B4 7ET, UK.

^5^ Sincrotrone Trieste, 34149 Basovizza, Trieste, Italy.

^6^ School of Science, RMIT University, Melbourne, VIC3001, Australia.

***** Correspondence: adam.lee2@rmit.edu.au; Tel.: +61-3-9925-2623

**Figure S1**: (left) N_2_ adsorption-desorption isotherms, and BJH pore size distributions for parent SBA-15, TiO_2_/SBA-15 and TiO_2_/MM-SBA-15. Type-IV isotherms with H1 hysteresis is characteristic of mesoporous SBA-15.

**Figure S2**: XRD patterns for the three prepared support materials, SBA-15, TiO_2_/SBA-15 and TiO_2_/MM-SBA-15.

**Table S1**: Elemental analysis of Ag/SBA-15, Ag/ TiO_2_/SBA-15 and Ag/TiO_2_/MM-SBA-15.

| **Material and nominal loading** | **Ag loading / wt%** | |
| --- | --- | --- |
|  | **Bulk^a^** | **Surface^b^** |
| 0.3 wt% Ag/SBA-15 | 0.3 | 0.45 |
| 1 wt% Ag/SBA-15 | 0.95 | 0.55 |
| 2 wt% Ag/SBA-15 | 2.3 | 1.1 |
| 0.3 wt% Ag/TiO_2_/SBA-15 | 0.3 | 0.55 |
| 1 wt% Ag/TiO_2_/SBA-15 | 0.9 | 0.65 |
| 2 wt% Ag/TiO_2_/SBA-15 | 2.3 | 1.4 |
| 0.3 wt% Ag/TiO_2_/MM-SBA-15 | 0.25 | 0.4 |
| 1 wt% Ag/TiO_2_/MM-SBA-15 | 0.75 | 0.7 |
| 2 wt% Ag/TiO_2_/MM-SBA-15 | 1.2 | 1.15 |

^a^ICP-OES, ^b^XPS

**Figure S3**: XRD patterns for (a) Ag/SBA-15, (b) Ag/TiO_2_/SBA-15, and (c) Ag/TiO_2_/MM-SBA-15 as a function of silver loading. All reflections associated with fcc silver metal.

**Figure S4**: Particle size distributions from TEM for (a) 0.3 wt%, (b) 0.95 wt% and (c) 2.4 wt% Ag nanoparticles deposited on SBA-15.

**Figure S5**: Particle size distributions from TEM (a) 0.3 wt%, (b) 0.9 wt% and (c) 2.4 wt% Ag nanoparticles deposited on TiO_2_/SBA-15.

**Figure S6**: Particle size distributions from TEM (a) 0.25 wt%, (b) 0.75 wt% and (c) 1.2 wt% Ag nanoparticles deposited on TiO_2_/MM-SBA-15.

**Figure S7**: Mean particle size and standard deviation for Ag/SBA-15, Ag/ TiO_2_/SBA-15, and Ag/TiO_2_/MM-SBA-15 as a function of bulk silver loading.

**Figure S8**: Fitted Ag 3d XP spectra of (a) Ag/SBA-15, (b) Ag/TiO_2_/SBA-15, and (c) Ag/TiO_2_/MM-SBA-15 as a function of Ag loading. All spectra fitted to carbonate and metal spin-orbit doublets possessing common lineshapes and fixed binding energies.

**Figure S9**: XANES profiles for (a) 0.3 wt%, (b) 0.95 wt% and (c) 2.4 wt% Ag/SBA-15.

**Figure S10**: XANES profiles for (a) 0.3 wt%, (b) 0.9 wt% and (c) 2.4 wt% Ag/TiO_2_/SBA-15.

**Figure S11**: XANES profiles for (a) 0.25 wt%, (b) 0.75 wt% and (c) 1.2 wt% Ag/TiO_2_/MM-SBA-15.

**Figure S12**: Fitted dissolution kinetics for Ag/SBA-15, Ag/TiO_2_/SBA-15, and Ag/TiO_2_/MM-SBA-15 as a function of particle size.

**Figure S13**: Logarithmic reductions for parent SBA-15, Ag/TiO_2_/SBA-15, and Ag/TiO_2_/MM-SBA-15 frameworks against (a) *Staphylococcus aureus* and (b) *Pseudomonas aeruginosa* after 24 h incubation.

**Figure S14**: Logarithmic reductions for (a) Ag/SBA-15, (b) Ag/TiO_2_/SBA-15, and (c) Ag/TiO_2_/MM-SBA-15 against *Staphylococcus aureus* and *Pseudomonas aeruginosa*.

**Figure S15**: Logarithmic colony forming units of (a) *S. aureus*, and (b) *P. aeruginosa* as a function of time for SBA-15, TiO_2_/SBA-15, and TiO_2_/MM-SBA-15.

**Table S2**: Ion concentrations in SBF solution.

| **Ion** | **Simulated Body Fluid / mM** | **Blood Plasma / mM** |
| --- | --- | --- |
| Na^+^ | 142 | 142 |
| K^+^ | 5 | 5 |
| Mg^2+^ | 1.5 | 1.5 |
| Ca^2+^ | 2.5 | 2.5 |
| Cl^-^ | 148.8 | 103 |
| HCO_3_^-^ | 4.2 | 27 |
| HPO_4_^2-^ | 1 | 1 |
| SO_4_^2-^ | 0.5 | 0.5 |
|  |  |  |
